# Optical and Electrical Properties of Al_x_Ga_1−x_N/GaN Epilayers Modulated by Aluminum Content

**DOI:** 10.3390/molecules29051152

**Published:** 2024-03-05

**Authors:** Wenwang Wei, Yanlian Yang, Yi Peng, Mudassar Maraj, Wenhong Sun

**Affiliations:** 1 Research Center for Optoelectronic Materials and Devices, School of Physical Science & Technology, Guangxi University, Nanning 530004, China; kindy789456@126.com (Y.Y.); pengyi41@outlook.com (Y.P.); mudassar@mail.ustc.edu.cn (M.M.); 2 Guangxi Key Laboratory of Calcium Carbonate Resources Comprehensive Utilization, College of Materials and Chemical Engineering, Hezhou University, Hezhou 542899, China

**Keywords:** AlGaN, HRXRD, XPS, photoluminescence, Hall effect

## Abstract

AlGaN-based LEDs are promising for many applications in deep ultraviolet fields, especially for water-purification projects, air sterilization, fluorescence sensing, etc. However, in order to realize these potentials, it is critical to understand the factors that influence the optical and electrical properties of the device. In this work, Al_x_Ga_1−x_N (x = 0.24, 0.34, 0.47) epilayers grown on c-plane patterned sapphire substrate with GaN template by the metal organic chemical vapor deposition (MOCVD). It is demonstrated that the increase of the aluminum content leads to the deterioration of the surface morphology and crystal quality of the AlGaN epitaxial layer. The dislocation densities of Al_x_Ga_1−x_N epilayers were determined from symmetric and asymmetric planes of the ω-scan rocking curve and the minimum value is 1.01 × 10^9^ cm^−2^. The $\left(10\bar{1}5\right)$ plane reciprocal space mapping was employed to measure the in-plane strain of the Al_x_Ga_1−x_N layers grown on GaN. The surface barrier heights of the Al_x_Ga_1−x_N samples derived from XPS are 1.57, 1.65, and 1.75 eV, respectively. The results of the bandgap obtained by PL spectroscopy are in good accordance with those of XRD. The Hall mobility and sheet electron concentration of the samples are successfully determined by preparing simple indium sphere electrodes.

## 1. Introduction

AlGaN is a ternary alloy with a direct band gap that may vary from 3.42 eV to 6.20 eV by adjusting the aluminum content, and it is widely used in ultraviolet (UV) photodetectors, light emitting diodes (LEDs) and laser diodes (LDs) [1,2,3,4]. AlGaN/GaN heterojunction materials exhibit strong voltage resistance, piezoelectric, and spontaneous polarization effects, which are conducted to the formation of high-density two-dimensional electron gas (2DEG), making them ideal materials for microwave power devices such as high electron mobility transistors (HEMTs) and heterojunction field effect transistors (HEFTs) [5,6,7]. Despite the immense potential of AlGaN materials, the presence of a high density of dislocations in AlGaN hinders the realization of high-performance AlGaN-based devices. Due to the absence of large-scale homogeneous epitaxial AlN substrates, heteroepitaxial growth of AlGaN materials using metal–organic chemical vapor deposition (MOCVD) has emerged as a widely adopted technique, leading to the formation of high-density dislocations within AlGaN epilayer. The presence of these defects and impurities could act as non-radiative recombination centers, resulting in reduced luminous efficiency of AlGaN/GaN multi-quantum Wells [8]. The epitaxial growth process of MOCVD is a highly intricate procedure, wherein alterations in the growth conditions such as temperature, rate, and carrier gas flow can significantly impact the migration ability of aluminum and gallium atoms, thereby influencing the surface morphology and interface quality of AlGaN/GaN. This may ultimately result in degradation in the photoelectric characteristics of the devices [9]. The threading dislocations (TDs) affect the early degradation of AlGaN/GaN high electron mobility sensor [10]. The electrons in the device bypass the gate control region through the defect clusters in the GaN buffer layer and undergo severe degradation [11]. As the aluminum content in the AlGaN buffer layer increases the dislocation density in the sample increases, which leads to a decrease in the two-dimensional electron gas (2DEG) mobility [12,13]. Nevertheless, AlGaN barriers containing a higher proportion of aluminum offer a significant conduction band discontinuity and an elevated Schottky barrier height, both contributing to enhanced device performance [14]. While the sheet carrier density may be augmented by raising the aluminum content in the ternary layer, the increased aluminum concentration adversely affects the quality of the AlGaN epitaxial layer [15,16]. In terms of theoretical study, some researchers have employed ab initio molecular dynamics simulations [17] and density functional thoery (DFT) calculations [18,19] to systematically investigate the growth mechanism and bandgap engineering of the ternary III-nitride material systems.

During the growth process, defects in AlGaN induce relaxation of tensile stress at the AlGaN/GaN interface, leading to a reduction in the incorporation rate of aluminum atoms and a significant decline for the mobility of 2DEG. This phenomenon also imposes limitations on achieving high crystallization quality for AlGaN films with elevated levels of aluminum content [20]. It is a great challenge for growing high-quality AlGaN with high aluminum content because of the large lattice mismatch and thermal expansion mismatch between sapphire substrate and epilayer, as well as the limited surface mobility of aluminum. The growth of GaN or AlN layers at elevated temperatures can thus be regarded as a viable approach for introducing strain relaxation layers, thereby enhancing the structural properties of nitride materials [21]. In addition, the crystalline quality of AlGaN/GaN heterojunction materials affects their electrical properties, which is closely related to the layer structure and growth process of the material [22,23,24,25]. Nitrogen-based device structures for electronic and optoelectronic applications typically contain Al_x_Ga_1−x_N layers, and n/p-type doping of these alloys is often required to enable precise control of the material’s electronic/optical properties and engineering applications [26,27]. Zhang et al. [28] reported a high-performance double heterojunction based AlGaN/GaN HEMT by incorporating a decreasing aluminum content graded AlGaN back barrier, which can suppress electron concentration in the buffer layer by avoiding forming parasitic channels. Chang et al. [29] demonstrated that the utilization of an AlGaN barrier, grown on a more compressive GaN layer, results in reduced tensile strain and improved surface morphology. Tao et al. [30] effectively reduced the dislocation density of AlGaN epilayer and improved the crystal quality of AlGaN by pretreating sapphire substrate with Al ion implantation. Nanopatterning technology is the most widely employed method in optoelectronic devices, which can effectively reduce the threading dislocation, obtain a smooth heterojunction interface, and improve the optical output. It can be seen that identifying and reducing the threading dislocation densities (TDDs) and internal stresses is crucial for optimizing the growth process of AlGaN/GaN epitaxial layers and improving device performance. Therefore, AlGaN/GaN heterojunction materials were grown on patterned sapphire substrate to improve the interface quality between AlGaN and GaN and reduce lattice defects by adjusting aluminum content so as to improve the optical and electrical properties of the device.

In this paper, investigation has been focused on Al_x_Ga_1−x_N (x = 0.24, 0.34, 0.47) epilayers grown on c-plane patterned sapphire substrate with GaN template by the metal organic chemical vapor deposition (MOCVD). Structural, morphological, optical and electrical properties have been analyzed and compared. Atomic force microscopy (AFM) and field emission scanning electron microscopy (SEM) have been performed to study morphology, thickness and crystalline quality. The content, in-plane strain, and threading dislocation densities of samples have estimated by high-resolution X-ray diffractometry (HRXRD). The chemical states are performed to determine by X-ray photoelectron spectroscopy (XPS) studies. The optical properties have been obtained by photoluminescence at room temperature, while electrical properties have been investigated by Hall measurements.

## 2. Results and Discussion

### 2.1. High Resolution X-ray Diffraction Study

**Figure 1:**
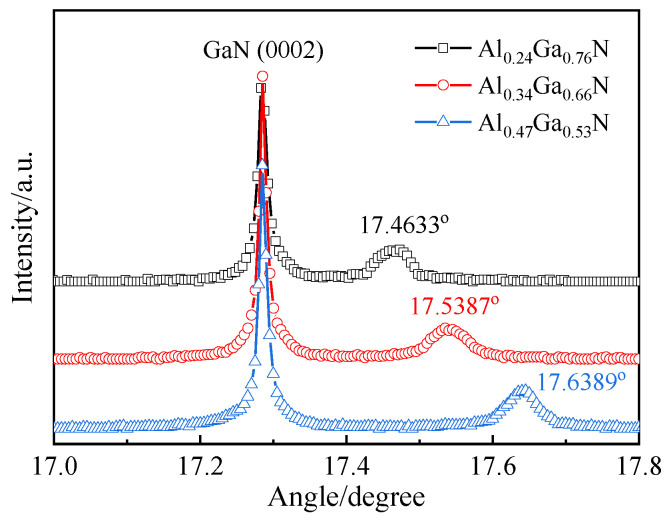
High resolution XRD ω-2θ scan of Al_x_Ga_1−x_N samples.

The ω-2θ scans of Al_x_Ga_1−x_N/GaN show the change of films content. The diffraction peaks for Al_x_Ga_1−x_N can be found by Lorentz fitting as 17.4633°, 17.5387°, and 17.6389°, as shown in Figure 1. The peak position of GaN buffer layer is a constant, which corresponds to the GaN (0002) diffraction plane. The interplanar spacing of the epilayers can be determined by Bragg′s law:

(1) $$d_{hkl}=\lambda /2\sin\theta \;$$

where *θ* is diffraction angle, *λ* is the X-ray wavelength and *d*_(*hkl*)_ is the distance between the crystal planes given by the Miller indices (*hkl*) [31]. The relationship between the interplanar spacing *d* along with (0001) orientation and the molar component x of ternary nitride alloy materials follows Vegard’s law:

(2) $$d\left({Al}_{x}{Ga}_{1-x}N\right)=x\cdot d\left(AlN\right)+\left(1-x\right)\cdot d\left(GaN\right)$$

where *d*(AlN) = 2.485 Å, *d*(GaN) = 2.593 Å [32]. The composition of the three samples can be confirmed as Al_0.24_Ga_0.76_N, Al_0.34_Ga_0.66_N and Al_0.47_Ga_0.53_N. In general, there is a bowing effect for the band gap of the nitride alloy. For Al_x_Ga_1−x_N semiconductor materials, the reported bowing constant *b* is ~0.69 eV. Therefore, the band gap *E*_g_ of Al_x_Ga_1−x_N as a function of Al content can be described as the equation:

(3) $$E_{g}\left({Al}_{x}{Ga}_{1-x}N\right)=x\cdot E_{g}\left(AlN\right)+\left(1-x\right)\cdot E_{g}\left(GaN\right)-b\cdot x\cdot \left(1-x\right)$$

where *E*_g_(GaN) and *E*_g_(AlN) denote the band gap values for GaN (3.42 eV) and AlN (6.20 eV), respectively [33,34]. The band gaps of Al_0.24_Ga_0.76_N, Al_0.34_Ga_0.66_N, and Al_0.47_Ga_0.53_N can be estimated to be 3.91 eV (317 nm), 4.15 eV (299 nm), and 4.48 eV (277 nm), respectively.

**Figure 2:**
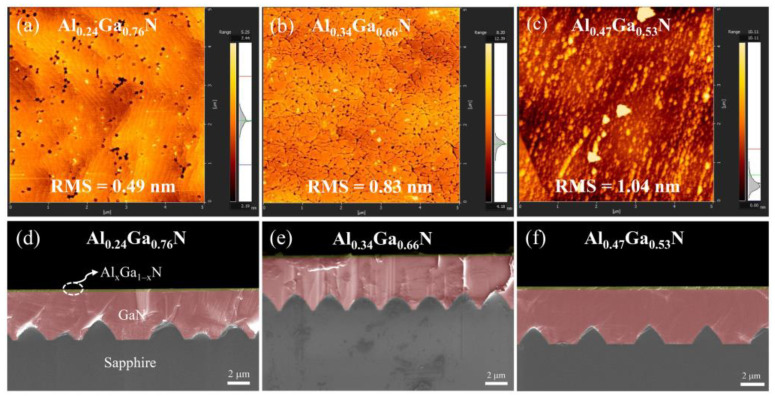
Atomic force microscopy (AFM) surface micrographs of samples: (**a**) Al_0.24_Ga_0.76_N and (**b**) Al_0.34_Ga_0.66_N and (**c**) Al_0.47_Ga_0.53_N, Scanning electron microscopy (SEM) cross-section images of samples: (**d**) Al_0.2_4Ga_0.76_N and (**e**) Al_0.34_Ga_0.66_N and (**f**) Al_0.47_Ga_0.53_N.

The surface morphology and thickness of Al_x_Ga_1−x_N layers have been observed by AFM and cross-sectional SEM, as demonstrated clearly in Figure 2. With the increase of Al component, the value of root mean square (RMS) surface roughness varies from 0.49, 0.83 and 1.04 nm. The bond between aluminum and nitrogen atoms is stronger compared to the Ga–N bond. A stronger bond can contribute to different physical and chemical properties, such as higher thermal and chemical stability. During the growth of AlGaN, the surface mobility of aluminum atoms is significantly lower than that of gallium. It is observed that aluminum atoms can migrate and create separate islands, a phenomenon attributed to the low surface mobility of aluminum atoms on the surface [9]. As the aluminum composition increased, the AlGaN growth was inhibited due to the low surface mobility of the aluminum species, leading to an increase in surface roughness. As a result, a deterioration in the crystal quality and surface morphology of the AlGaN epilayers was observed. This behavior is also manifested by an increased density of dislocations. The thickness of the AlGaN films in the three samples is 0.20 μm, 0.22 μm, and 0.26 μm, respectively, as indicated in Table 1.

The full width at half maximum (FWHM) of X-ray rocking curves (XRC) diffraction patterns serves as an indirect indicator of various types of threading dislocation densities. Specifically, the FWHM of symmetric (0002) diffraction is particularly responsive to pure screw-type threading dislocations, whereas the FWHM of asymmetric diffraction provides an effective measure of pure edge-type threading dislocations. The TDDs of the Al_x_Ga_1−x_N epilayers have been estimated using the equation [35]:

(4) $$\rho =\beta^{2}/\left(4.35\times b^{2}\right)$$

where *ρ* represents dislocation density, *β* stands for FWHM of XRC, and *b* is the Burgers vector length (*b*_screw_ = *c*_AlxGa1−xN_, *b*_edge_ = *a*_AlxGa1−xN_ [36]. The screw, edge, and mixed types of the TDDs for Al_x_Ga_1−x_N epilayers have been calculated and presented in Table 1. The edge dislocation density is one order of magnitude larger than that of the screw and play a domination role in Al_x_Ga_1−x_N epilayers. As the Al composition increase the TDDs are noted to change from 1.01 × 10^9^ cm^−2^ to 3.09 × 10^9^ cm^−2^, indicating that more TDs are formed with the higher Al composition Al_x_Ga_1−x_N layers. Thus, the density of dislocations increases, resulting in a rougher surface morphology for these samples.

Nitride epilayers are described as crystals with a mosaic structure that can be characterized by means of tilt and twist angles. A set of important parameters, such as lateral coherence length *L*_//_, vertical coherence length *L*_⊥_, dislocation tilt angle *β*_t_, and non-uniform strain $\varepsilon_{in}^{⊥}$ are obtained by the Williamson–Hall method [37]. Formulas (5) and (6) are applicable for ω-scanning and ω-2θ scanning of symmetric triaxial crystal diffraction on the (000l) crystal plane, respectively.

(5) $$\beta_{\omega }\frac{sin\theta }{\lambda }=\frac{1}{2L_{∕∕}}+\beta_{t}sin\theta /\lambda$$

(6) $$\beta_{\omega -2\theta }\frac{cos\theta }{\lambda }=\frac{1}{2L_{⊥}}+\varepsilon_{in}^{⊥}sin\theta /\lambda$$

where *β*_ω_ and *β*_ω-2θ_ represent the peak of FWHM of ω-scan and ω-2θ scan, respectively, *θ* is the Bragg angle and *λ* is the wavelength. The variable of *L*_//_, *L*_⊥_, *β*_t_ and $\varepsilon_{in}^{⊥}$ can be obtained by graphing the linear relationship between Formulas (5) and (6).

**Figure 3:**
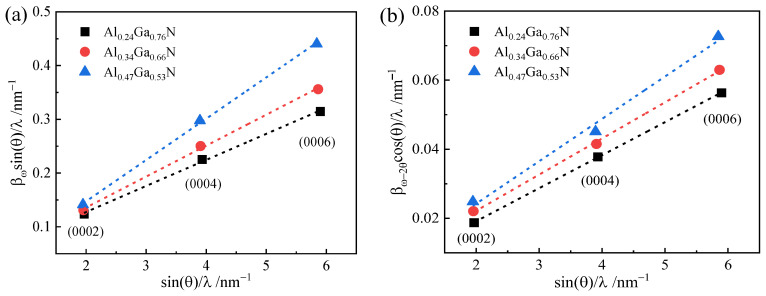
Williamson–Hall plots of AlGaN epilayers for symmetric reflections: (**a**) ω-scan and (**b**) ω-2θ scan. The dotted lines result from a linear fit of data.

Figure 3 shows the Williamson–Hall plot for the Al_x_Ga_1−x_N epilayers of various Al composition, where ω-scans and ω-2θ scans have been measured for three symmetric reflections: (0002), (0004) and (0006). The corresponding parameters of Al_x_Ga_1−x_N epilayers are deduced by linear fitting from Figure 3, as listed in Table 1. From the table, it can be observed that the lateral coherence length *L*_//_ and tilt angle *β*_t_ increase with increase of Al composition in the Al_x_Ga_1−x_N epilayers. The tilt angle generated by dislocation varies from 0.0485° to 0.0768°, indicating that the TDs in the AlGaN epilayers increase with increasing Al fraction. The vertical coherence length increases with the epilayer thickness from 0.163 to 0.251 μm. In case of micro-strain in the direction of growth shows a direct proportion to epilayer’s thickness and Al composition.

The “*c*” lattice constant can be determined by measuring the (004) reflection in 2θ scan, utilizing the relationship between d-spacing and a general (*hkl*) reflection for hexagonal crystal structures. The value of the “*a*” lattice can be obtained from a 2θ scan of the (105) reflection, using the given equation [38]: $\frac{1}{d^{2}}=\frac{4\left(h^{2}+hk+k^{2}\right)}{3a^{2}}+\frac{l^{2}}{c^{2}}\;$. While the in-plane strain values of GaN epilayer extracted from equation $\varepsilon_{a}=\frac{a-a_{0}}{a_{0}}\;$. The parameter “*a*” represents the measured lattice value of GaN, while “*a*_0_” denotes the nominal value of GaN film in its fully relaxed state [24]. The in-plane strain values of GaN epilayer can be calculated to be −2.14 × 10^−3^.

**Figure 4:**
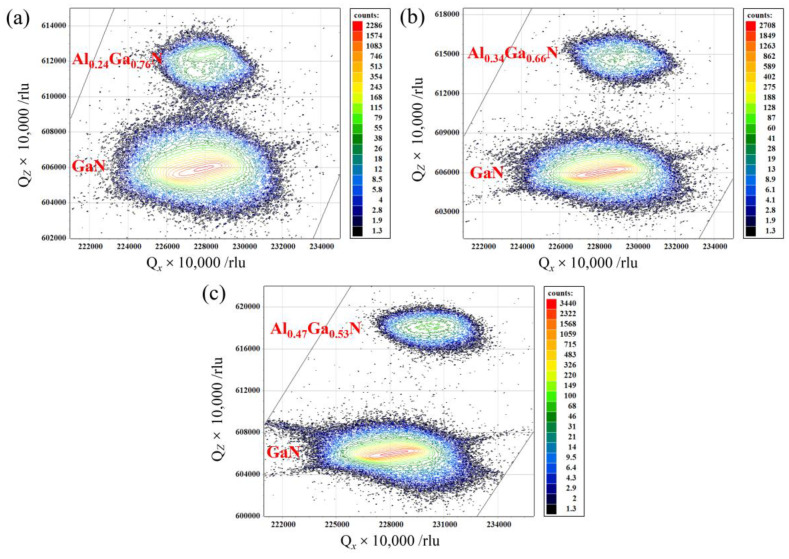
Reciprocal space mapping of $\left(10\bar{1}5\right)$ reflection for samples: (**a**) Al_0.24_Ga_0.76_N, (**b**) Al_0.34_Ga_0.66_N and (**c**) Al_0.47_Ga_0.53_N.

The reciprocal space mapping (RSM) is a two-dimensional measurement technique that the shape and positions of the reciprocal lattice points or intensity contour plots can reveal important information, such as mismatch, strain state, relaxation, defects, and chemical composition, etc. The nominal Al compositions have been found to be ~24%, ~34% and ~47% for Al_x_Ga_1−x_N epilayers. Figure 4 shows the $\left(10\bar{1}5\right)$ RSM of Al_x_Ga_1−x_N/GaN heterostructure epilayers. The results demonstrate that as the Al composition increases, the maximum reflection intensity of Al_x_Ga_1−x_N reciprocal lattice points gradually shifts from a fully strained state towards a partially relaxed state. Due to its thinner thickness compared to the Al_x_Ga_1−x_N layer, the GaN layer exhibits a lower peak intensity in reflection. The in-plane strain *ε*_xx_ for the Al_x_Ga_1−x_N/GaN hetero-epilayers have been estimated by using the equation [36]:

(7) $$\varepsilon_{xx}=q_{x}^{GaN}/q_{x}^{Epi}-1$$

where $q_{x}^{GaN}$ and $q_{x}^{Epi}$ denote the x positions of the GaN and the AlGaN layer to be determined, respectively. The reciprocal lattice units (rlu) in RSM represent a fraction relative to the lattice constant in reciprocal space. When a crystal has a lattice constant of *a* Å, the relationship between them can be expressed as 1 rlu = 2π/*a* Å^−1^. It can be deduced that the in-plane strain *ε*_xx_ is −3.34 × 10^−4^, −3.46 × 10^−3^, and −8.10 × 10^−3^ for Al_0.24_Ga_0.76_N, Al_0.34_Ga_0.66_N and Al_0.47_Ga_0.53_N samples, respectively, implying the presence of partially strain between the GaN and Al_x_Ga_1−x_N epilayers. The in-plane strain in the epilayers increase with increasing Al composition. Arivazhagan et al. [24] have studied that the AlGaN/GaN heterostructure at 14% Al composition has zero in-plane strain value. Feng et al. [39] determined the overall in-plane strain *ε_a_* = (*a* − *a*_0_)/*a*_0_ and out-of-plane strain *ε*_c_ = (*c* − *c*_0_)/*c*_0_ in the Al_x_Ga_1−x_N layers, and discovered that the biaxial stress and strain within the Al_x_Ga_1−x_N/AlN heterostructures exhibit an increasing trend with higher Al content, and the c-plane of the Al_x_Ga_1−x_N epilayer experiences compressive strain while the a-plane undergoes tensile strain.

### 2.2. X-ray Photoelectron Spectroscopy Study

**Figure 5:**
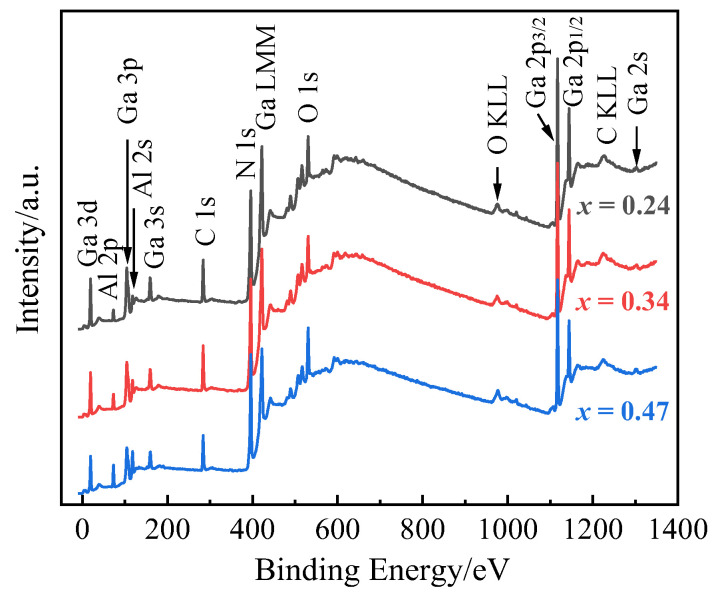
The XPS wide-scan spectra of three Al_x_Ga_1−x_N/GaN heterostructures.

To further investigate the structural and chemical states on the surface of the Al_x_Ga_1−x_N epilayers, XPS was conducted. The XPS wide-scan spectra of the Al_x_Ga_1−x_N/GaN heterostructures with different Al content are shown in Figure 5, indicating the presence of the elements C, N, O, Al and Ga. The intense photoelectron 3d, 3p, 2p, and Auger LMM peaks are observed for Ga, in addition to 1s peak for C, N, and O. The C 1s peak is resulted by the ambient carbon or impurities adsorbed on the sample surface. The smaller intensity peaks corresponding to Al 2p, Al 2s, and Ga 3s are also observed. The intensity of Al 2s and 2p peaks increase as the increase of Al content.

**Figure 6:**
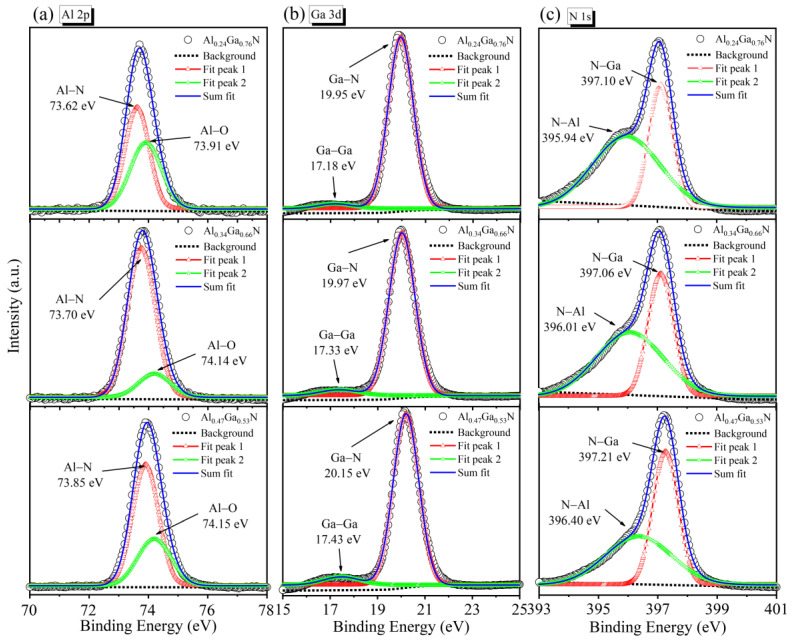
The XPS fine-scan spectra fitting results for Al_x_Ga_1−x_N/GaN heterostructures: (**a**) Al 2p, (**b**) Ga 3d and (**c**) N 1s.

The fine scans of the Al 2p, Ga 3d, and N 1s core level peaks are performed for three samples and displayed in Figure 6a–c. In Figure 6a, the Al 2p core level spectrum is deconvoluted into two sub-peaks, which can be assigned to Al–Al and Al–N bonding for three samples (Al_0.24_Ga_0.76_N, Al_0.34_Ga_0.66_N, Al_0.47_Ga_0.53_N). The binding energies of Al–N are 73.62, 73.70, and 73.85 eV; the binding energies of Al–O are 73.91, 74.14, and 74.15 eV, respectively. The sample is minimally oxidized at x = 0.34. The Ga 3d peaks can be separated into two components, at 17.18–17.43 eV, related to Ga–Ga bonding; the strong peak at 19.95–20.15 eV corresponds to Ga–N bonding, as shown in Figure 6b. It can be seen that there is a small peak corresponding to the metallic Ga in the sample, indicating the presence of residual gallium. Deconvolutions of the N 1s peak for the Al_x_Ga_1−x_N samples with different Al content are compared in Figure 6c and show the bonding of N–Al and N–Ga. The binding energies of N–Al and N–Ga show an increase from 395.94 to 396.40 eV and from 397.10 to 396.40 eV, respectively. These results indicate that the Al 2p, Ga 3d, and N 1s core level of Al_x_Ga_1−x_N epilayers have shifted towards higher binding energy with increment in Al content. The binding energy of forming the same chemical bond is related to the ratio of elemental components in the film. Charge transfer causes a change in binding energy, in addition to other factors such as electric fields, hybridization, and ambient charge density [40].

**Figure 7:**
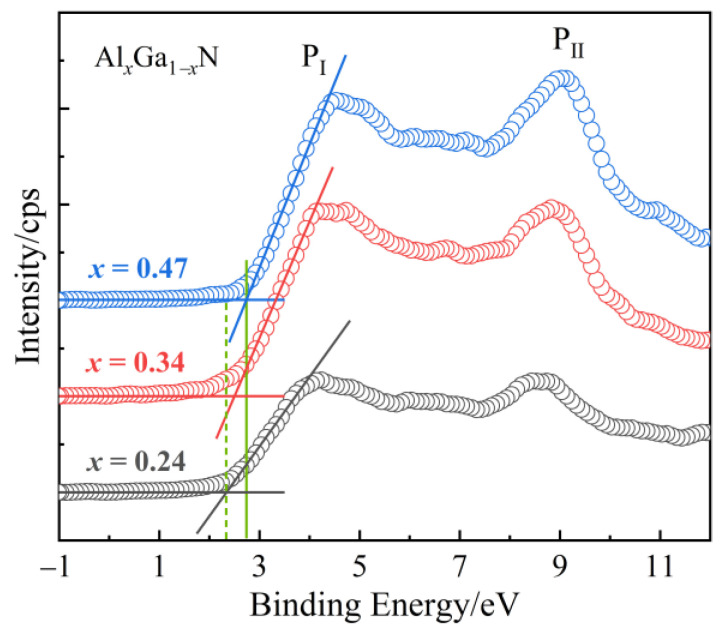
High resolution XPS valence band spectra of the Al_x_Ga_1−x_N samples.

The XPS valence band (VB) spectra of the Al_x_Ga_1−x_N samples are represented in Figure 7. The valence states are split into two sub-band labelled as P_I_ and P_II_ located at ~4.4 and ~8.9 eV, respectively. The density maximum of N states of p-symmetry located in P_II_ along with Al d and p states have the same energy. The ratio of intensity of the two peaks (P_II_/P_I_) increases with increasing the Al content in the Al_x_Ga_1−x_N epilayers. It is mainly the different hybridization between d and p states for cation and anion in the nitride. For cation, the Al 4d and N p states are more strongly hybridized than that of Ga 4d state. The VB maximum is 2.34, 2.50, and 2.73 eV for Al_0.24_Ga_0.76_N, Al_0.34_Ga_0.66_N, and Al_0.47_Ga_0.53_N, respectively, presenting a movement away from the valence band with Al content. The surface barrier height, which is defined as the energy separation between conduction band minimum and Fermi level was calculated to 1.57, 1.65, and 1.75 eV for Al_0.24_Ga_0.76_N, Al_0.34_Ga_0.66_N, and Al_0.47_Ga_0.53_N, respectively. For as grown AlGaN surface, the surface barrier height dependence of film thickness and Al content indicate that the existence of low-density and distributed surface donor states.

### 2.3. Photoluminescence Study

**Figure 8:**
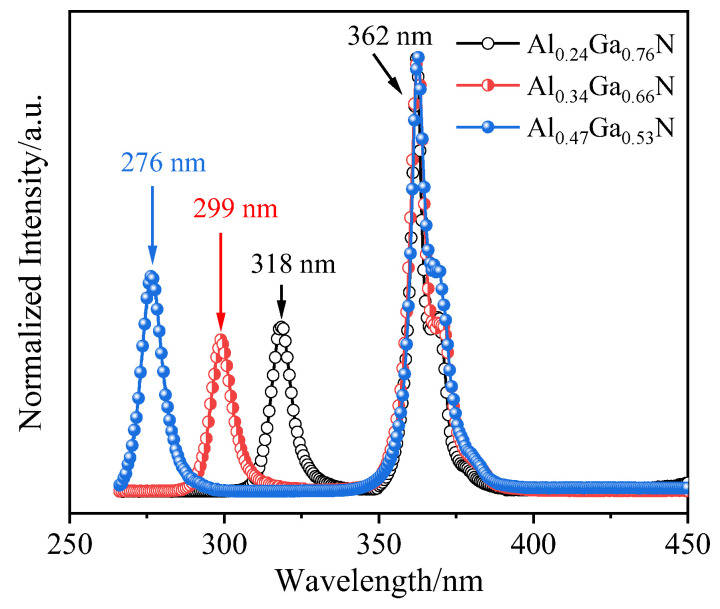
Room temperature PL emission spectra of Al_x_Ga_1−x_N/GaN.

Figure 8 shows three Al_x_Ga_1−x_N/GaN samples photoluminescence (PL) as a function of wavelength at room temperature. The PL emission of Al_0.24_Ga_0.76_N, Al_0.34_Ga_0.66_N, and Al_0.47_Ga_0.53_N layers are 318 nm (3.90 eV), 299 nm (4.15 eV), and 276 nm (4.49 eV), respectively, which are excellently consistent with XRD results. The PL intensity exhibits slight variations, while FWHM increases from 6.2 to 8.9 nm with an increase in the aluminum content of Al_x_Ga_1−x_N epilayers. The narrower FWHM indicate the Al_x_Ga_1−x_N layers have the better crystal quality. This may be due to the fact that the surface migration of Al atom is much lower than that of Ga atom. And the nucleation growth is inhibited with the increase of Al component, leading to the decrease of crystal quality. The emission peak around 3.42 eV is observed in each PL spectrum, corresponding to a wavelength of 362 nm, which is attributed to band-edge emission of GaN. In AlGaN/GaN heterostructures, these built-in polarization fields can induce quantum confined Stark effect, resulting in a shift and broadening of the emission peak. This effect is caused by the separation of electron and hole wavefunctions within the quantum wells, which is attributed to internal electric fields. The separation reduces the overlap between wavefunctions, thereby impacting recombination efficiency and causing a shift in emission wavelength. Additionally, these polarization fields can lead to a decrease in oscillator strength, potentially contributing to changes in PL intensity independent of crystal quality.

In nitride materials, the threading dislocations act as deep-level impurities and non-radiative centers, and the intensity of near-band edge emission is greatly dependent on the dislocations in the epitaxial layer [41]. This result has been discovered to corroborate the structural quality of Al_x_Ga_1−x_N epilayer and is in well agreement with the results of HRXRD.

### 2.4. Hall Effect Measurements

**Figure 9:**
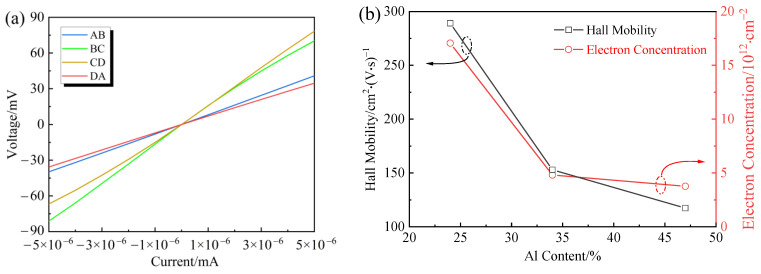
(**a**) Current–voltage characteristic curve for Al_0.24_Ga_0.76_N sample, (**b**) Hall mobility and sheet electron concentration of Al_x_Ga_1−x_N/GaN samples.

Hall effect measurements were conducted to investigate the influence of Al content on the electrical properties of Al_x_Ga_1−x_N epilayers which equipped with pure indium electrode (99.99%) on the hot plate around 230 °C during 3 min. The I–V characteristic curve satisfying the Ohmic contact is shown in Figure 9a. The carrier mobility of Al_0.24_Ga_0.76_N, Al_0.34_Ga_0.66_N, and Al_0.47_Ga_0.53_N layers grown by MOCVD are 289.14, 152.94, and 117.34 cm^2^/V∙s, respectively. The carrier mobility and sheet electron concentration of Al_x_Ga_1−x_N samples are shown in Figure 9b. It can be seen that Hall mobility and sheet electron concentration decrease with the increase of Al content. The relationship between sheet resistance and Hall mobility can be mathematically described by $R_{s}=\frac{1}{qn\mu }$. Where *q* represents the charge quantity, *n* denotes the sheet electron concentration, and *μ* signifies the Hall mobility. It should be noted that the sheet resistance exhibits an inverse proportionality to the sheet electron concentration. Thus, the value of sheet resistance (*R_s_*) can be calculated to be 1262, 8502, and 14,376 Ω/sq for three samples. The study conducted by Jena et al. [42] reveals that dislocation scattering serves as a dominant scattering mechanism limiting the mobility of 2DEGs characterized by high dislocation densities. There are several possible explanations for the lower carrier mobility in this result. (1) Electron mobility in semiconductor structures like AlGaN/GaN 2DEGs is influenced by various scattering mechanisms, not just dislocation scattering. Other factors include interface roughness, impurity scattering, phonon scattering, and alloy disorder scattering. The actual mobility is a result of the interplay between these different mechanisms. (2) While a high dislocation density can significantly reduce mobility due to increased scattering sites, a density of 10^9^ cm^−2^ might not be sufficient alone to lower the mobility to the observed levels. This suggests that other scattering mechanisms are also significantly contributing. (3) The quality of the AlGaN/GaN interfaces and the overall crystal quality can have a major impact on mobility. Imperfections, defects, and interface roughness can all contribute to additional scattering, reducing mobility. (4) Mobility is also temperature-dependent. At higher temperatures, phonon scattering becomes more significant, which can reduce the mobility. A comprehensive analysis considering all potential scattering sources and their interactions is essential to fully understand and optimize electron mobility in these materials.

The growth of superior crystals and the enhancement of thin film properties have consistently been the focus of attention. Arivazhagan et al. [24] investigated the structural and electrical characteristics of Al_x_Ga_1–x_N/GaN (x = 0.14, 0.26, 0.45) epitaxially grown on flat sapphire substrate by MOCVD. The lowest value 1.3 × 10^9^ cm^–2^ of dislocation density was found at 26% Al content. But the AlGaN layer with Al content of 14% has been observed to exhibit a zero in-plane strain value, indicating pseudomorphic growth. Both Meng et al. [25] and Luong et al. [29] studied on the dislocation density and carrier mobility of AlGaN/GaN structures containing 25% aluminum. Upon comparison, it was observed that Meng’s sample exhibited higher dislocation density and increased carrier mobility. The implication is that the decrease in 2EDG mobility does not solely result from scattering caused by high dislocation density but may also involve synergistic effects of other mechanisms. The structural and morphological properties of Al_x_Ga_1–x_N (x = 0.15, 0.20, 0.33, 0.51) epilayers with GaN template have been studied by Loganathan et al. [41]. The results showed that the growth rate of AlGaN decreased with the increase of Al composition. The influence of dislocation density on the transport properties of AlGaN/GaN high electron mobility transistor (HEMT) structures was reported by Hájek et al. [43]. By comparison, it can be found that under the same conditions, the carrier mobility of the AlGaN with 24% Al grown on the flat sapphire is 1360 cm^2^/V·s, while that is only 539 cm^2^/V·s on the patterned sapphire. It showed experimentally that lowering the dislocation density considerably increases the electron mobility in 2DEG. As compared to Al_x_Ga_1–x_N/GaN grown on flat sapphire substrate, the dislocation density, optical, and electrical parameters of the Al_x_Ga_1−x_N/GaN heterostructures grown on patterned sapphire substrate have been given in this study. It is evident that the utilization of patterned sapphire substrates can effectively mitigate dislocation density in AlGaN epitaxial structures. However, it should be noted that the carrier mobility of Al_x_Ga_1−x_N/GaN heterojunctions may be compromised. The abovementioned details are summarized in Table 2.

## 3. Materials and Methods

The three Al_x_Ga_1−x_N/GaN samples were grown on a 2-inch diameter, 430-μm-thick c-plane patterned sapphire substrates by Aixtron 200/4 RF-S MOCVD (Aixtron, Herzogenrath, Germany) system with trimethylgallium (TMGa), trimethylaluminum (TMAl), and ammonia (NH_3_) as Ga, Al, and N sources, respectively. High pure hydrogen (H_2_) was used as carrier gas. First, the patterned sapphire substrates for all samples were thermally cleaned in H_2_ ambient for 10 min, then the nitriding pretreatment was carried out for 60 s with a nitrogen flow rate of 5000 sccm and a temperature of 700 °C. Second, a thin low temperature GaN buffer layer was deposited at 525 °C, under a growth pressure of 550 torr with a V/III flux ratio of 12000, and the deposition thickness was 0.2 μm. Third, a 4.3-µm thick high temperature GaN template (RSM = 0.50 nm), with a growth pressure of 550 torr and a V/III flux ratio of 6500, was grown at 1060 °C. Finally, the 0.20–0.26 μm thick AlGaN epilayers were grown at 1060 °C with the TMAl flow rate of 20.5–52.3 μmol/min, and reactor pressure was varied to grow three AlGaN samples with three Al contents (24%, 34%, and 47%), other growth conditions kept unchanged.

The surface and cross-sectional morphology of the Al_x_Ga_1−x_N samples were characterized by atomic force microscopy (Hitachi, Tokyo, Japan) and field emission scanning electron microscopy (Hitachi, Tokyo, Japan). High-resolution X-ray diffractometry (Malvern PANalytical, Alemlo, The Netherlands) equipped with Ge (220) four-crystal monochromator and utilizing Cu Kα1 radiation with a wavelength of 1.5406 Å was employed for the X-ray measurements. This technique was used to analyze the composition and stress in the Al_x_Ga_1−x_N epitaxial layers. Additionally, the densities of screw-type and edge-type dislocations were estimated using the (0002) and $\left(10\bar{1}2\right)$ reflections observed in the XRC. The chemical states and valence band were identified by X-ray photoelectron spectroscopy (Thermo Fisher Scientific, Waltham, MA, USA) with a monochromatic Al Kα radiation of 1486.6 eV. The energy resolution of this setup was approximately 0.6 eV, and the base pressure in the sample chamber was maintained below 7.0 × 10^−9^ mbar. The binding energies of all samples were calibrated by referencing the C 1s core line at 284.8 eV. The XPSPEAK 4.1 software containing a Voigt mixture of Gauss–Lorentz function and Shirley model that were performed to fit and analysis data. The photoluminescence measurements (HORIBA Jobin Yvon, Paris, France) were conducted under 261 nm laser light excitation. Hall measurements (Ecopia, Anyang, Republic of Korea) were used to investigate carrier mobility, electron concentration and sheet resistance. All measurements were performed at room temperature.

## 4. Conclusions

In conclusion, the surface morphology and crystal quality of Al_x_Ga_1−x_N/GaN epilayers are affected by the Al content. The bandgaps of Al_0.24_Ga_0.76_N, Al_0.34_Ga_0.66_N, and Al_0.47_Ga_0.53_N were determined using XRD, yielding values of 3.91 eV, 4.15 eV, and 4.48 eV, respectively. As the Al content increases, the growth of the film is hindered by the low surface mobility of Al atoms, resulting in an increase in sample surface roughness with RMS values of 0.49 nm, 0.83 nm, and 1.04 nm, respectively. The dislocation density, with the minimum value at 1.01 × 10^9^ cm^−2^, is mainly modulated by the Al content as observed by both Williamson–Hall method and XRC, in case of high Al content samples exhibit greater dislocation density and in-plane strain. The in-plane strains *ε*_xx_ of the Al_x_Ga_1−x_N samples are −3.34 × 10^−4^, −3.46 × 10^−3^, and −8.10 × 10^−3^, respectively, indicating a partial strain between GaN and Al_x_Ga_1−x_N epilayers. The surface barrier heights of the Al_x_Ga_1−x_N samples are 1.57, 1.65, and 1.75 eV, respectively. The results of the bandgap width and crystal quality obtained by PL spectroscopy are in good accordance with those of XRD. With the increase in Al content, there is a slight change in the PL intensity of the Al_x_Ga_1−x_N epilayer, accompanied by a variation in the FWHM of PL peak from 6.2 to 8.9 nm, indicating a gradual deterioration in the crystal quality of the Al_x_Ga_1−x_N layer. Finally, the electrical properties of the samples are successfully determined by preparing simple indium sphere electrodes. These studies are important for the further preparation and development of the performance of AlGaN-based LEDs.

## Figures and Tables

**Table 1:** The parameters of Williamson–Hall plots and threading dislocation density for high temperature GaN layer and Al_x_Ga_1−x_N samples.

Sample No.	Thickness (μm)	*L*_//_ (μm)	*β*_t_ (°)	*L*_⊥_ (μm)	$\varepsilon_{in}^{⊥}$	Screw Dislocation Densities (10^8^ cm^−2^)	Edge Dislocation Densities (10^9^ cm^−2^)	Mixed Dislocation Densities (10^9^ cm^−2^)	RSM (nm)
GaN	4.30	0.216	0.0217	0.135	0.0583	0.96	0.32	0.42	0.50
Al_0.24_Ga_0.76_N	0.20	0.145	0.0485	0.163	0.0957	1.31	0.88	1.01	0.49
Al_0.34_Ga_0.66_N	0.22	0.403	0.0575	0.204	0.1046	1.83	1.53	1.71	0.83
Al_0.47_Ga_0.53_N	0.26	0.664	0.0768	0.251	0.1228	2.40	2.85	3.09	1.04

**Table 2:** Comparison of dislocation density, optical and electrical parameters of the Al_x_Ga_1−x_N/GaN heterostructures grown on flat sapphire substrate (FSS) and patterned sapphire substrate (PSS) by MOCVD method.

Type of Substrate	Al Content	Screw Dislocation Density (cm^−2^)	Edge Dislocation Density (cm^−2^)	Root Mean Square (nm)	PL Peaks (eV)	In-Plane Strain *ε_xx_*	Sheet Carrier Density (cm^−2^)	Mobility (cm^2^/V·s)	Ref.
FSS	11%	5.87 × 10^7^	5.89 × 10^8^	0.26	3.66	–	–	–	[8]
FSS	14%	1.9 × 10^8^	2.5 × 10^9^	0.34	3.68	0.000	–	–	[24]
	26%	2.5 × 10^8^	1.0 × 10^9^	0.36	3.93	−8.37 × 10^−4^	–	–	
	45%	5.2 × 10^8^	6.2 × 10^9^	0.81	4.43	−1.53 × 10^−2^	–	–	
FSS	25%	3.51 × 10^8^	3.65 × 10^9^	0.402	–	–	1.02 × 10^13^	1508	[25]
FSS	25%	1.81 × 10^8^	1.37 × 10^9^	0.63	–	–	1.45 × 10^13^	1300	[29]
FSS	45%	2.10 × 10^8^	3.70 × 10^8^	0.176	–	−1.80 × 10^−3^	–	–	[36]
FSS	15%	2.42 × 10^8^	2.718 × 10^9^	0.26	3.73	–	–	–	[41]
	20%	2.91 × 10^8^	3.053 × 10^9^	0.49	3.81	–	–	–	
	33%	4.22 × 10^8^	5.102 × 10^9^	1.17	4.09	–	–	–	
	51%	5.43 × 10^8^	5.881 × 10^9^	1.48	4.45	–	–	–	
FSS	24%	9.36 × 10^8^		–	–	–	1.56 × 10^13^	1360	[43]
FSS	30%	1.35 × 10^8^	6.98 × 10^9^	0.845	–	–	–	–	[44]
PSS	24%	6.81 × 10^8^		–	–	–	10.90 × 10^13^	539	[43]
PSS	24%	1.31 × 10^8^	0.88 × 10^9^	0.49	3.90	−3.34 × 10^−4^	1.71 × 10^13^	289.14	This work
	34%	1.83 × 10^8^	1.53 × 10^9^	0.83	4.15	−3.46 × 10^−3^	0.48 × 10^13^	152.94	
	47%	2.40 × 10^8^	2.85 × 10^9^	1.04	4.49	−8.10 × 10^−3^	0.37 × 10^13^	117.34	

## Data Availability

Data are contained within the article.

## References

[1] Pandit B., Schubert E.F., Cho J. (2020). Dual-functional ultraviolet photodetector with graphene electrodes on AlGaN/GaN heterostructure. Sci. Rep..

[2] Lucie V., Vincent G., Sylvain F., Catherine B., Joël E., Gwénolé J., Christophe D. (2023). M-plane AlGaN digital alloy for microwire UV-B LEDs. Appl. Phys. Lett..

[3] Khaouani M., Hamdoune A., Bencherif H., Kourdi Z., Dehimi L. (2020). An ultra-sensitive AlGaN/AlN/GaN/AlGaN photodetector: Proposal and investigation. Optik.

[4] Daqing P., Zhonghui L., Chuanhao L., Qiankun Y., Dongguo Z., Weike L., Xun D. (2023). Low stress AlGaN/GaN heterojuction with AlGaN buffer grown on 6-inch semi-insulating SiC substrate. J. Cryst. Growth.

[5] Xue D., Zhang H., Liu J., Xia X., Guo W., Huang H., Xu N., Xi Q., Liang H. (2021). Improved performance of AlGaN/GaN HEMT based H+ sensors by surface hydroxylation treatment. Mater. Sci. Semicond. Process.

[6] Xiaobiao H., Wang L., Qiliang W., Shaoheng C., Liuan L., Liang H. (2023). Design of normally-off p-GaN/AlGaN/GaN heterojunction field-effect transistors with re-grown AlGaN barrier. J. Cryst. Growth.

[7] Li L., Yamaguchi R., Wakejima A. (2020). Polarization engineering via InAlN/AlGaN heterostructures for demonstration of normally-off AlGaN channel field effect transistors. Appl. Phys. Lett..

[8] Hou Y., Wang B., Yang J., Zhang y., Zhang Z., Liang F., Liu Z., Zhao D. (2023). Influence of growth interruption on the morphology and luminescence properties of AlGaN/GaN ultraviolet multi-quantum wells. Opt. Express.

[9] Tang L., Tang B., Zhang H., Yuan Y. (2020). Review—Review of Research on AlGaN MOCVD Growth. ECS J. Solid State Sci. Technol..

[10] Gu L., Yang S., Miao B., Gu Z., Wang J., Sun W., Wu D., Li J. (2019). Electrical detection of trace zinc ions with an extended gate-AlGaN/GaN high electron mobility sensor. Analyst.

[11] Ma Z., Cao H., Lin S., Li X., Zhao L. (2019). Degradation and failure mechanism of AlGaN-based UVC-LEDs. Solid-State Electron..

[12] Narang K., Bag R.K., Singh V.K., Pandey A., Saini S.K., Khan R., Arora A., Padmavati M.V.G., Tyagi R., Singh R. (2020). Improvement in surface morphology and 2DEG properties of AlGaN/GaN HEMT. J. Alloys Compd..

[13] Khan M.A.K., Alim M.A., Gaquiere C. (2021). 2DEG transport properties over temperature for AlGaN/GaN HEMT and AlGaN/InGaN/GaN pHEMT. Microelectron. Eng..

[14] Sun Y., Wang Y., Tang J., Wang W., Huang Y., Kuang X. (2019). A Breakdown Enhanced AlGaN/GaN Schottky Barrier Diode with the T-Anode Position Deep into the Bottom Buffer Layer. Micromachines.

[15] Kaun S.W., Wong M.H., Mishra U.K., Speck J.S. (2012). Correlation between threading dislocation density and sheet resistance of AlGaN/AlN/GaN heterostructures grown by plasma-assisted molecular beam epitaxy. Appl. Phys. Lett..

[16] Jiyu Z., Xiaobo L., Taofei P., Yue H., Xiao W., Yuyu B., Liuan L., Jin-Ping A. (2021). Surface sensibility and stability of AlGaN/GaN ion-sensitive field-effect transistors with high Al-content AlGaN barrier layer. Appl. Surf. Sci..

[17] Kakanakova-Georgieva A., Ivanov I.G., Suwannaharn N., Hsu C.-W., Cora I., Pécz B., Giannazzo F., Sangiovanni D.G., Gueorguiev G.K. (2021). MOCVD of AlN on epitaxial graphene at extreme temperatures. CrystEngComm.

[18] Alves Machado Filho M., Hsiao C.L., Dos Santos R.B., Hultman L., Birch J., Gueorguiev G.K. (2023). Self-Induced Core-Shell InAlN Nanorods: Formation and Stability Unraveled by Ab Initio Simulations. ACS Nanosci Au.

[19] Zhang Q., Li Q., Zhang W., Zhang H., Zheng F., Zhang M., Hu P., Wang M., Tian Z., Li Y. (2022). Phase transition and bandgap engineering in B_1-x_Al_x_N alloys: DFT calculations and experiments. Appl. Surf. Sci..

[20] Ajayan J., Nirmal D., Mohankumar P., Mounika B., Bhattacharya S., Tayal S., Fletcher A.S.A. (2022). Challenges in material processing and reliability issues in AlGaN/GaN HEMTs on silicon wafers for future RF power electronics & switching applications: A critical review. Mater. Sci. Semicond. Process..

[21] Xu R., Kang Q., Zhang Y., Zhang X., Zhang Z. (2023). Research Progress of AlGaN-Based Deep Ultraviolet Light-Emitting Diodes. Micromachines.

[22] Mishra M., Gupta G. (2020). Electronic properties and oxygen chemisorption at Al_x_Ga_1−x_N surfaces. Mater. Chem. Phys..

[23] Kim H., Yun H.J., Choi S., Choi B.J. (2020). Comparison of electrical and interfacial characteristics between atomic-layer-deposited AlN and AlGaN on a GaN substrate. Appl. Phys. A.

[24] Arivazhagan P., Ramesh R., Kumar R.R., Faulques E., Bennis F., Baskar K. (2016). Structural and electrical characteristics of GaN, n-GaN and Al_x_Ga_1−x_N. J. Alloys Compd..

[25] Meng Q., Lin Q., Jing W., Mao Q., Zhao L., Fang X., Dong T., Jiang Z. (2021). Characterization of the Electrical Properties of a Double Heterostructure GaN/AlGaN Epitaxial Layer with an AlGaN Interlayer. J. Electron. Mater..

[26] Kakanakova-Georgieva A., Nilsson D., Stattin M., Forsberg U., Haglund Å., Larsson A., Janzén E. (2010). Mg-doped Al_0.85_Ga_0.15_N layers grown by hot-wall MOCVD with low resistivity at room temperature. Phys. Status Solidi (RRL) Rapid Res. Lett..

[27] Van de Walle C.G., Stampfl C., Neugebauer J., McCluskey M.D., Johnson N.M. (1999). Doping of AlGaN Alloys. MRS Internet J. Nitride Semicond. Res..

[28] Zhang H., Sun Y., Hu K., Yang L., Liang K., Xing Z., Wang H., Zhang M., Yu H., Fang S. (2023). Boosted high-temperature electrical characteristics of AlGaN/GaN HEMTs with rationally designed compositionally graded AlGaN back barriers. Sci. China Inf. Sci..

[29] Luong T.-T., Tran B.T., Ho Y.-T., Ha M.-T.-H., Hsiao Y.-L., Liu S.-C., Chiu Y.-S., Chang E.-Y. (2015). Performance improvements of AlGaN/GaN HEMTs by strain modification and unintentional carbon incorporation. Electron. Mater. Lett..

[30] Tao H., Xu S., Su H., Zhang T., Zhang J., Zhang Y., Gao Y., Liu X., Lu H., Xie L. (2023). Improved crystal quality of AlGaN by Al ion-implantation sapphire substrate. Mater. Lett..

[31] Wang S., Zhang X., Yang H., Cui Y. (2015). Effect of fluctuation in Al incorporation on the microstructure, bond lengths, and surface properties of an Al_x_Ga_1−x_N epitaxial layer. Electron. Mater. Lett..

[32] Xu Y.-N., Ching W. (1993). Electronic, optical, and structural properties of some wurtzite crystals. Phys. Rev. B.

[33] Monemar B., Bergman J., Buyanova I. (2021). GaN and Related Materials: Optical Characterisation of GaN and Related Materials.

[34] Yamashita H., Fukui K., Misawa S., Yoshida S. (1979). Optical properties of AlN epitaxial thin films in the vacuum ultraviolet region. J. Appl. Phys..

[35] Wei W., Peng Y., Yang Y., Xiao K., Maraj M., Yang J., Wang Y., Sun W. (2022). Study of Defects and Nano-patterned Substrate Regulation Mechanism in AlN Epilayers. Nanomaterials.

[36] Arivazhagan P., Ramesh R., Jayasakthi M., Loganathan R., Balaji M., Baskar K. (2013). Structural and carrier dynamics of GaN and AlGaN-based double heterostructures in the UV region. J. Electron. Mater..

[37] Nemoz M., Dagher R., Matta S., Michon A., Vennéguès P., Brault J. (2017). Dislocation densities reduction in MBE-grown AlN thin films by high-temperature annealing. J. Cryst. Growth.

[38] Wallis D.J., Zhu D., Oehler F., Westwater S.P., Pujol A., Humphreys C.J. (2013). Measuring the composition of AlGaN layers in GaN based structures grown on 150 mm Si substrates using (2 0 5) reciprocal space maps. Semicond. Sci. Technol..

[39] Feng Y., Saravade V., Chung T.-F., Dong Y., Zhou H., Kucukgok B., Ferguson I.T., Lu N. (2019). Strain-stress study of Al_x_Ga_1−x_N/AlN heterostructures on c-plane sapphire and related optical properties. Sci. Rep..

[40] Yin J., Chen D., Yang H., Liu Y., Talwar D.N., He T., Ferguson I.T., He K., Wan L., Feng Z.C. (2021). Comparative spectroscopic studies of MOCVD grown AlN films on Al_2_O_3_ and 6H–SiC. J. Alloys Compd..

[41] Loganathan R., Jayasakthi M., Prabakaran K., Ramesh R., Arivazhagan P., Baskar K. (2014). Studies on dislocation and surface morphology of Al_x_Ga_1−x_N/GaN heterostructures grown by MOCVD. J. Alloys Compd..

[42] Jena D., Gossard A.C., Mishra U.K. (2000). Dislocation scattering in a two-dimensional electron gas. Appl. Phys. Lett..

[43] Hájek F., Hospodková A., Hubík P., Gedeonová Z., Hubáček T., Pangrác J., Kuldová K. (2021). Transport properties of AlGaN/GaN HEMT structures with back barrier: Impact of dislocation density and improved design. Semicond. Sci. Technol..

[44] Lu L., Shen B., Xu F., Gao B., Huang S., Miao Z., Qin Z., Yang Z., Zhang G., Zhang X. (2008). Morphology and microstructure evolution of Al_x_Ga_1−x_N epilayers grown on GaN/sapphire templates with AlN interlayers observed by transmission electron microscopy. J. Appl. Phys..

